# Two new species of Scymnini (Coleoptera: Coccinellidae) from Karnataka, India

**DOI:** 10.3897/BDJ.3.e5296

**Published:** 2015-06-22

**Authors:** J. Poorani

**Affiliations:** ‡ ICAR-National Bureau of Agricultural Insect Resources, P.B. No. 2491, HA Farm Post, Bellary Road, Hebbal, Bangalore 560024, India

**Keywords:** Coleoptera, Coccinellidae, *
Horniolus
*, *
Scymnus
*, new species, India

## Abstract

**Background:**

The Scymnini (Coleoptera: Coccinellidae) of the Indian region is rich and highly speciose, with nearly 90 described species and scores of undescribed species (Poorani 2002). There is a dire need to systematically revise the genera and species of this tribe from the Indian region. Due to paucity of representative collections covering the entire region and lack of access to types, it is difficult to identify most of the Scymnini of the Indian region to species. As a result, many economically important species remain poorly characterized, or worse, unnamed.

**New information:**

Two economically important and unique species of Scymnini (Coccinellidae) belonging to *Horniolus* Weise (1900) and Scymnus (Pullus) Mulsant (1846) from the Southern Indian state of Karnataka that have remained unnamed for long are treated in this paper. These species are externally similar to other known species and often misidentified. *Horniolus sororius* sp. n. and Scymnus (Pullus) rajeshwariae sp. n. (Coleoptera: Coccinellidae) are described here and illustrated with notes on their biology and related species.

## Introduction

This paper is an effort to give names to two unique species belonging to *Horniolus* Weise (1900) and Scymnus (Pullus) Mulsant (1846) that are found in the southern Indian state of Karnataka. These two genera have been traditionally placed in the tribe Scymnini under the subfamily Scymninae, both of which are no longer recognized. Under the new system of classification proposed for Coccinellidae by Seago et al. (2011) based on molecular phylogeny, Scymnini is treated as part of the composite tribe Coccidulini under the subfamily Coccinellinae. The species of *Horniolus* described here has been commonly misidentified as *H. guimeti* (Mulsant), Scymnus (Pullus) latemaculatus Motschulsky, and *Pharoscymnus horni* (Weise) in view of their external similarity. It is a general predator of mealybugs on coffee and other plants. The second species is a highly host-specific predator found in association with the bamboo woolly aphid, *Pseudoregma bambusicola* (Takahashi) (Hemiptera: Aphididae: Hormaphidinae) and has not been collected anywhere else from India in over fifteen years of collection. These two species are described and illustrated here.

## Materials and methods

The specimens examined in this study were collected from the southern Indian state of Karnataka during the course of my studies on Coccinellidae of southern India over several years (1998-2014). A few specimens came from the collections of the erstwhile Commonwealth Institute of Biological Control-Indian Station, Bangalore (now National Bureau of Agricultural Insect Resources, Bangalore). For preparation of male and female genitalia, whole specimens were immersed in warm soapy water for 10–20 minutes depending on their age. The abdomen was gently detached with a minuten pin and kept overnight in 10% KOH. The genitalia were dissected in distilled water and transferred to glycerol for studies and imaging. After examination, the genitalia were transferred to microvials and pinned beneath the respective specimens. The following measurements were made using the measurement module of a Leica M205A stereo microscope: total length, from apical margin of clypeus to apex of elytra (TL); total width, across both elytra at their widest point (TW=EW); pronotal length, from the middle of anterior margin to the base of pronotum (PL); pronotal width at its widest (PW); elytral length along suture from apex to base including scutellum (EL). Images of immature stages were taken with a Nikon D7000 DSLR camera. Images of whole specimens and their diagnostic characters were taken using a Leica DFC 420 camera attached to a Leica M205A stereo microscope. Composite images were generated from image stacks using Combine ZP and touched up in Adobe Photoshop Elements 11. The specimens studied are deposited in the following collections:

NBAIR – National Bureau of Agricultural Insect Resources, Bangalore

UASB – University of Agricultural Sciences, Bangalore

## Taxon treatments

### Scymnus (Pullus) rajeshwariae

Poorani sp. n.

urn:lsid:zoobank.org:act:3FF45295-D9C9-4D94-9C56-FFF8159A2B08

#### Materials

- **Type status:** Holotype. **Occurrence:** sex: male; preparations: whole animal; **Location:** continent: Asia; country: India; stateProvince: Karnataka; municipality: Bangalore; locality: Hebbal; verbatimCoordinates: 13°01'N, 77°35'E; **Event:** eventDate: 2002-01-01; eventRemarks: Feeding on *Pseudoregma bambusicola* on bamboo
- **Type status:** Paratype. **Occurrence:** sex: 7 males, 8 females; preparations: whole animal; **Location:** continent: Asia; country: India; stateProvince: Karnataka; municipality: Bangalore; locality: Hebbal; verbatimCoordinates: 13°01'N, 77°35'E; **Event:** eventDate: 2002-01-01; eventRemarks: Feeding on *Pseudoregma bambusicola* on bamboo

#### Description

Length: 2.50–2.90 mm; Width: 1.70–2.10 mm; TL/TW: 1.33–1.40; PL/PW: 0.52–0.58; EL/EW: 0.97–1.05; EW/PW: 1.34–1.43. **Male**: Body outline (Fig. 1e) elongate oval, broadest around middle of elytra, elytra apically somewhat broadly rounded to subtruncate; dorsum densely pubescent with silvery white hairs. Head dark pitchy brown to black, anterior clypeal margin slightly lighter; pronotum black except anterior and lateral margins narrowly reddish testaceous; elytra black, apical one-fourth reddish brown-testaceous. Ventral side with mouthparts, legs and abdominal ventrites dark brown to testaceous, middle of abdominal ventrite 1 and remaining areas dark pitchy brown. Head with interocular distance about 1.7x as wide as an eye; punctures dense in posterior half, slightly more widely spaced towards clypeal margin, separated by <0.5–1 diameter. Antenna 11-segmented with a distinct club. Pronotum with punctures on disc shallow, separated by 1–4 diameters, denser and more closely spaced on lateral sides, separated by <1–2 diameters. Elytra with shallowly impressed punctures, slightly larger and denser than that on pronotum, separated by 1–2 diameters on disc, slightly coarser, denser and more closely spaced around anterolateral margins, interspaces between punctures coriaceous; with a row of slightly larger punctures on either side of suture in anterior half. Prosternal process (Fig. 2a) with a pair of apically divergent carinae. Abdominal postcoxal lines (Fig. 2c) complete, broadly semicircular to boat-shaped, area enclosed densely punctate in anterior half, punctures coarser and fewer in posterior half, apical one-fourth adjacent to postcoxal line more or less smooth, devoid of punctures. Ventrite 5 with posterior margin weakly emarginate, ventrite 6 truncate. Tarsi pseudotrimerous. Tarsal claws bifid with a basal tooth (Fig. 2b). Male genitalia (Fig. 2d, e, f) with penis guide lanceolate in ventral view (Fig. 2e), parameres shorter than penis guide in lateral view (Fig. 2d), bilobate with triangular / subconical inner expansions in anterior half, inner expansion much shorter than outer, with long apical hairs reaching beyond apex of outer lobe, apices of outer lobes of parameres with much longer, denser hairs reaching beyond apex of penis guide. Penis (Fig. 2f) coiled with a prominent capsule having a distinctly longer outer arm than inner arm, penis ­­­apex membranous with a short, hook-like projection.

**Female**: Similar to male. Tarsal claws (Fig. 2b) with a more transverse basal tooth than in male, anterior process distinctly shorter than posterior (in male basal tooth more distinctly quadrate, posterior process only slightly longer than anterior). Last abdominal ventrites not showing any marked dimorphism, ventrite 5 with posterior margin broadly arcuate, ventrite 6 subtruncate. Female genitalia (Fig. 1f) with spermatheca having well differentiated cornu, nodulus and ramus and a sclerotised rod-like projection on bursa.

#### Diagnosis

Scores of Oriental species of Scymnini are externally similar to S. (P.) rajeshwariae sp. n. in having black elytra with reddish brown / testaceous apices and are difficult to identify without examination of male genitalia. But the habits and male genitalia of this species appear to be unique. The male genitalia are very distinctive with bilobed parameres and the penis guide is apically lanceolate.

#### Etymology

This species is named for Ms. S.K. Rajeshwari, Technical Officer, NBAIR, who has been a great help and support in my work on Coccinellidae.

#### Distribution

India: Karnataka.

#### Biology

It appears to be a highly specific predator of the bamboo woolly aphid, *Pseudoregma bambusicola* (Takahashi) and is collected always in association with this species only. The larvae are greyish with white waxy dusting and lack the usual dense waxy filaments in many other *Scymnus* spp. (Fig. 1a, b, c​). The larvae are commonly seen in aphid colonies on bamboo stem, but the adults are more cryptic in their habits and found feeding only under the leaf sheaths. There are several common predators of *P. bambusicola* which occur along with this species. These include the giant bamboo ladybirds, *Synonycha grandis* (Thunberg) and *Megalocaria dilatata* (F.) (Coccinellidae), *Dipha aphidivora* (Meyrick) (Lepidoptera: Pyralidae), and *Dideopsis aegrota* (F.) (Diptera: Syrphidae). In view of its specificity, this species could be a potential biological control agent of *P. bambusicola* on bamboo.

### Horniolus sororius

Poorani sp. n.

urn:lsid:zoobank.org:act:738B0BE1-73C8-477E-9C1A-3475CB9C3930

#### Materials

- **Type status:** Holotype. **Location:** continent: Asia; country: India; stateProvince: Karnataka; locality: Balehonnur; verbatimLocality: Central Coffee Research Station; **Event:** year: 1999; habitat: Coffee plantation; **Record Level:** institutionID: ICAR-National Bureau of Agricultural Insect Resources; institutionCode: NBAIR; ownerInstitutionCode: NBAIR
- **Type status:** Paratype. **Occurrence:** recordedBy: P.K. Vinod Kumar; individualCount: 6; **Location:** continent: Asia; country: India; stateProvince: Karnataka; locality: Central Coffee Research Station, Balehonnur; **Identification:** identifiedBy: J. Poorani; **Event:** eventDate: 1999; year: 1999; habitat: coffee plantation; **Record Level:** institutionID: NBAIR; institutionCode: NBAIR; ownerInstitutionCode: NBAIR
- **Type status:** Paratype. **Occurrence:** recordedBy: Sunil Joshi; individualCount: 1; **Location:** continent: Asia; country: India; stateProvince: Karnataka; locality: PDBC, Bangalore; **Identification:** identifiedBy: J. Poorani; **Event:** eventDate: vi.2002; year: 2002; habitat: on wing; **Record Level:** institutionID: NBAIR; institutionCode: NBAIR; ownerInstitutionCode: NBAIR
- **Type status:** Paratype. **Occurrence:** recordedBy: L. Lakshmi; individualCount: 1; **Location:** continent: Asia; country: India; stateProvince: Karnataka; locality: Hessarghatta, Bangalore; **Identification:** identifiedBy: J. Poorani; **Event:** eventDate: 3.vi.2009; year: 2009; **Record Level:** institutionID: NBAIR; institutionCode: NBAIR; ownerInstitutionCode: NBAIR
- **Type status:** Other material. **Occurrence:** individualCount: 1; sex: female; **Location:** country: India; stateProvince: Karnataka; locality: Bangalore; **Event:** eventDate: 1958-12-11; year: 1958; month: 12; day: 11; verbatimEventDate: 11XII1958; habitat: Resting on *Eleusine coracana*; **Record Level:** institutionID: NBAIR; datasetID: CIBC-BS
- **Type status:** Other material. **Occurrence:** individualCount: 1; sex: female; **Location:** continent: Asia; country: India; stateProvince: Karnataka; locality: Hebbal, Bangalore; **Event:** eventDate: 2001-09; year: 2001; month: 09; day: 09; verbatimEventDate: 09092001; habitat: Feeding on *Aleurodicus dispersus*; **Record Level:** institutionID: NBAIR; datasetID: CIBC-BS

#### Description

Length: 2.20-2.65 mm; width: 1.65–1.80 mm. **Male**: Body (Fig. 3) elongate oval, moderately convex. Head and pronotum testaceous to dark brown, elytra dark brown with four yellowish spots – first pair transverse, roughly quadrate, located before middle in anterior half, second pair smaller, transverse, located around 4/5^th^ of elytra, both spots occasionally larger, almost touching lateral margins of elytra; head with elongate silvery white hairs, pronotum with yellowish white to brownish pubescence, elytra with a mixture of dark brown and yellowish white hairs, those on elytral spots paler, those on rest of elytra predominantly brown to black. Ventral side more or less uniform reddish brown, pro-, meso- and metasternites darker reddish brown, tarsi of legs slightly lighter, yellowish brown. Head with clypeal margin carinate, punctures separated by 2-3 diameters, denser and separated by less than their own diameter near eye margins. Pronotum densely punctate on posterior and lateral margins, slightly more widely spaced on disc, separated by 3-5 diameters. Elytral punctures denser and slightly larger than those on pronotum, separated by 2-5 diameters on disc, punctures on lateral sides and apices more closely spaced. Prosternal process with an inverted Y-shaped carina. Abdominal postcoxal line (Fig. 4a) complete and semicircular, area enclosed by postcoxal line sparsely punctate with few punctures along anterior margins. Ventrite 5 truncate, ventrite 6 barely emarginate. Tarsi trimerous. Male genitalia (Fig. 4b, c, d, e) with tegmen in ventral view (Fig. 4b, c) with penis guide shorter than parameres, apically very slightly asymmetrical, broadest at base, progressively narrowed to apex, apical one-fifth triangular; parameres longer than penis guide, apices with elongate hairs. Penis capsule (Fig. 4d) with a prominent inner arm, outer arm lacking; apex of penis (Fig. 4e) strongly flattened or spatulate, densely spotted. **Female**: Similar to male. Ventrite 6 apically weakly arcuate. Genitalia with spermatheca (Fig. 4f) with spermatheca tubular, long and intricately coiled, sperm duct elongate and progressively broader.

#### Diagnosis

The external appearance of *H. sororius* sp. n. is very similar to that of *Horniolus vietnamicus* Miyatake. *Horniolus hisamatsui* Miyatake also has a similar elytral pattern, but it has reddish brown head and pronotum. But the male genitalia, particularly the enlarged siphonal apex, are diagnostic and unique to this species. *Horniolus sororius* sp. n. is also similar to two common Indian species, Scymnus (Pullus) latemaculatus Motschulsky (Fig. 5a), and *Pharoscymnus horni* (Weise) (Fig. 5d). The former is close to *H. sororius* sp. n. in general appearance, but can be distinguished by the presence of parallel, apically divergent carinae on the prosternal process and the male genitalia (Fig. 5b, c) are also diagnostic. Besides, it is one of the most common general predators of aphids in India. *Pharoscymnus horni* can be differentiated from *H. sororius* sp. n. by its distinctly more rounded body outline and other generic characters.

#### Etymology

The specific epithet is a Latin adjective (“sororius” L.= of a sister, sisterly) in reference to its similarity to other common species.

#### Distribution

India: Karnataka.

#### Biology

Collected in association with coffee mealybugs and on *Eleusine coracana* (label data). This species was recorded by Ramani et al. (2002) (as *Horniolus* sp.) as a predator of spiralling whitefly, *Aleurodicus dispersus* Russell (Hemiptera: Aleyrodidae).

#### Notes

*Horniolus* is an Oriental genus and only three species, *H. dispar* Weise (1900) (from Sri Lanka), *H. nigripes* Miyatake (1976) (from southern India) and *H. hisamatsui* (Nepal) are represented in the Indian subcontinent. *Horniolus guimeti* (Mulsant), distributed in Malaysia and Borneo, has been mentioned in literature from the Indian subcontinent, but is a doubtful record for India (R.G. Booth, BMNH, *in litt*.). Korschefsky (1931) includes India in its distribution range, but Indian records of *H. guimeti* are mostly misidentifications of Scymnus (Pullus) latemaculatus (Fig. 5a), and *Pharoscymnus horni*, which are externally similar.

## Supplementary Material

XML Treatment for Scymnus (Pullus) rajeshwariae

XML Treatment for Horniolus sororius

## Figures and Tables

**Figure 1a.**
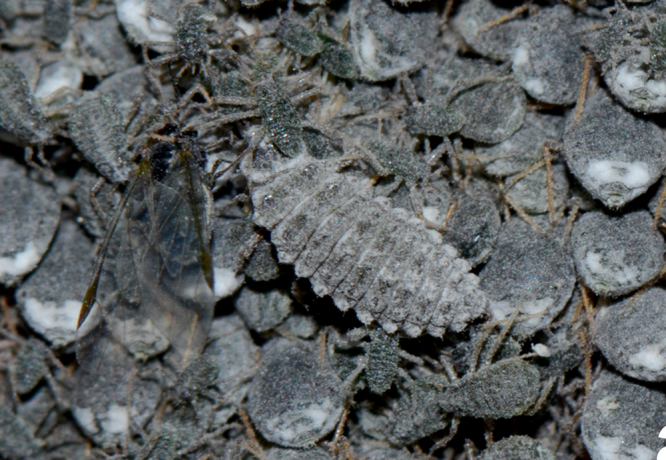
Larva feeding on *Pseudoregma bambusicola* on bamboo

**Figure 1b.**
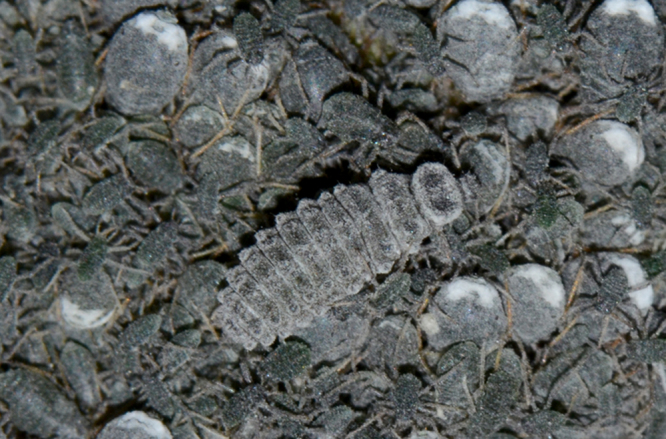
Larva feeding on *Pseudoregma bambusicola* ​on bamboo

**Figure 1c.**
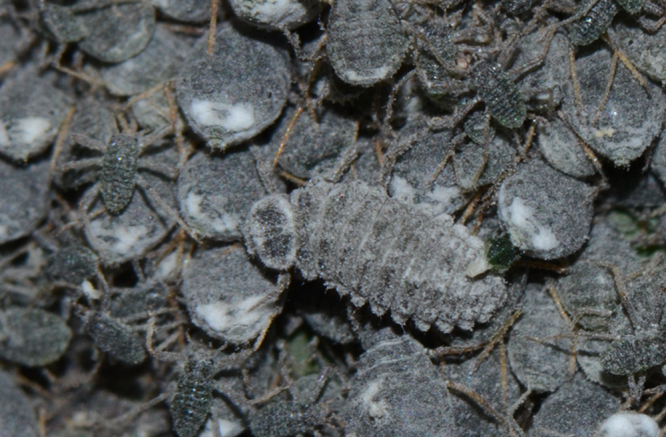
Larva feeding on *Pseudoregma bambusicola* ​on bamboo

**Figure 1d.**
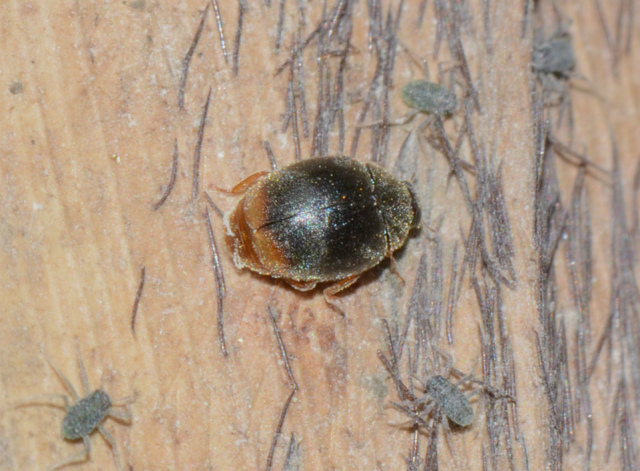
Adult feeding on *Pseudoregma bambusicola* ​on bamboo

**Figure 1e.**
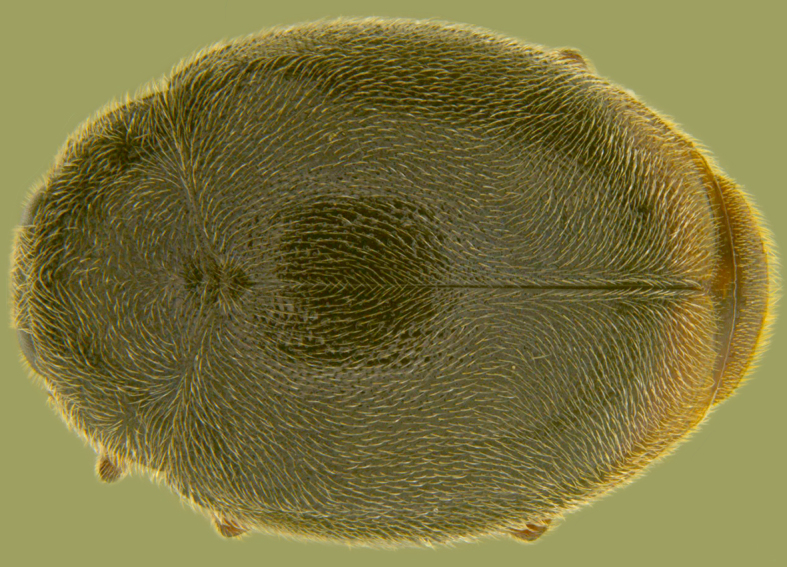
Adult, dorsal view

**Figure 1f.**
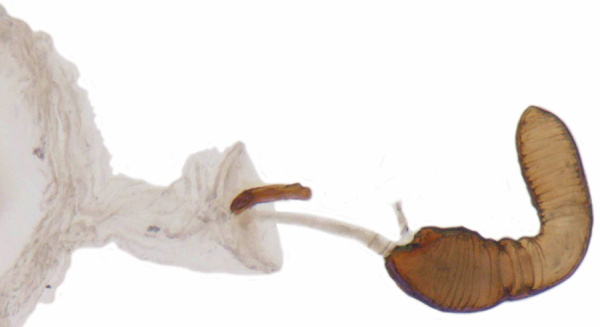
Female genitalia: Spermatheca

**Figure 2a.**
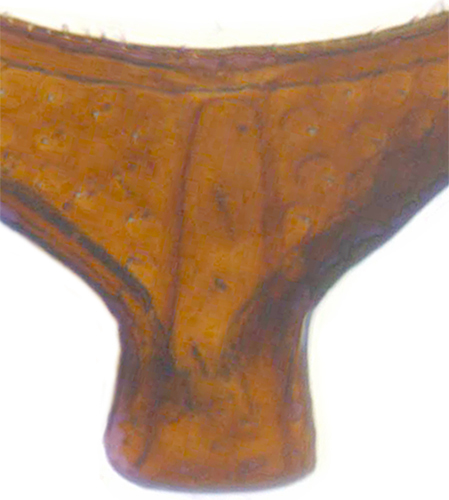
Prosternal process

**Figure 2b.**
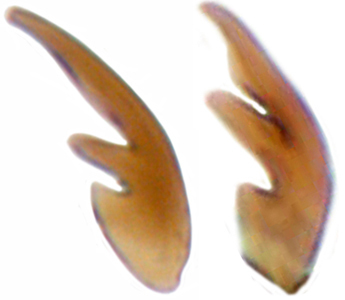
Tarsal claw in female (left) and male (right)

**Figure 2c.**
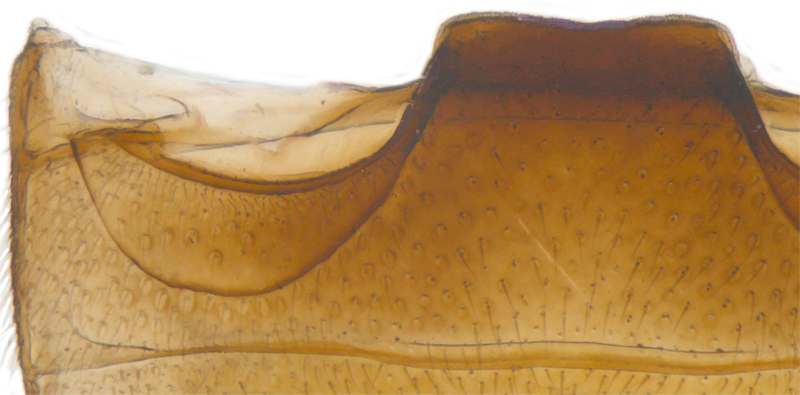
Abdominal postcoxal line

**Figure 2d.**
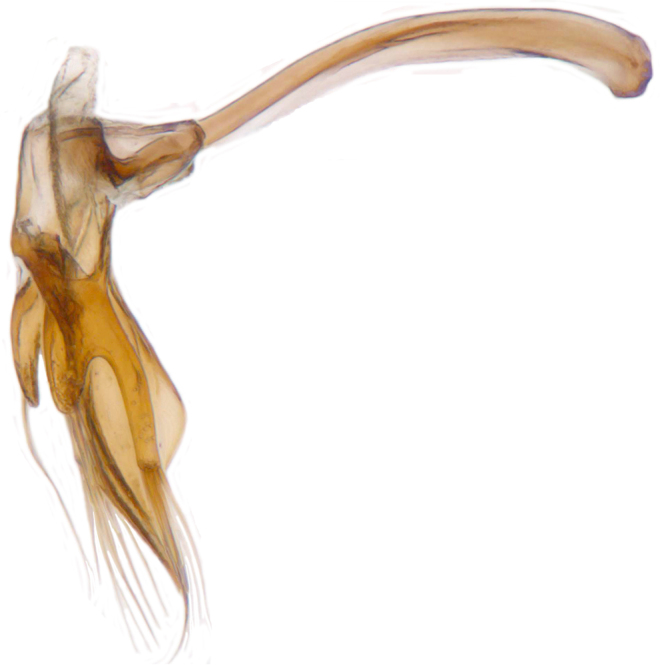
Male genitalia: Tegmen, lateral view

**Figure 2e.**
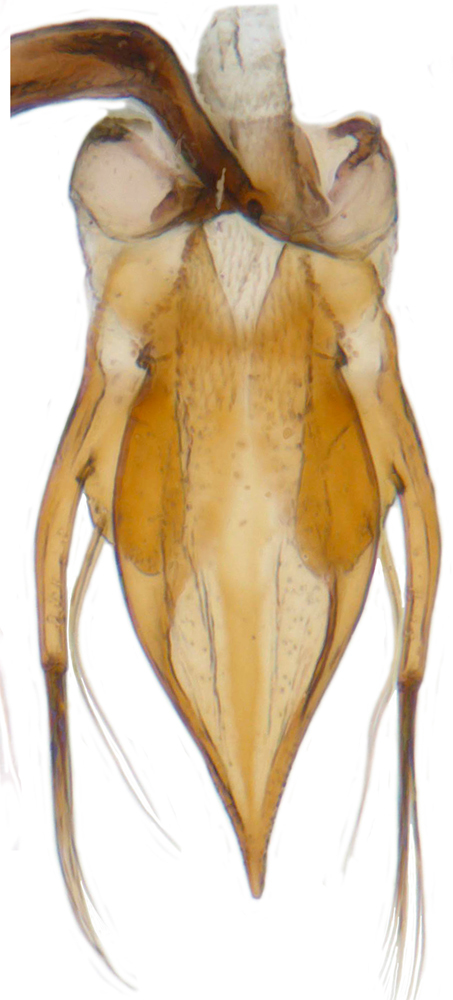
Male genitalia: Tegmen, ventral view

**Figure 2f.**
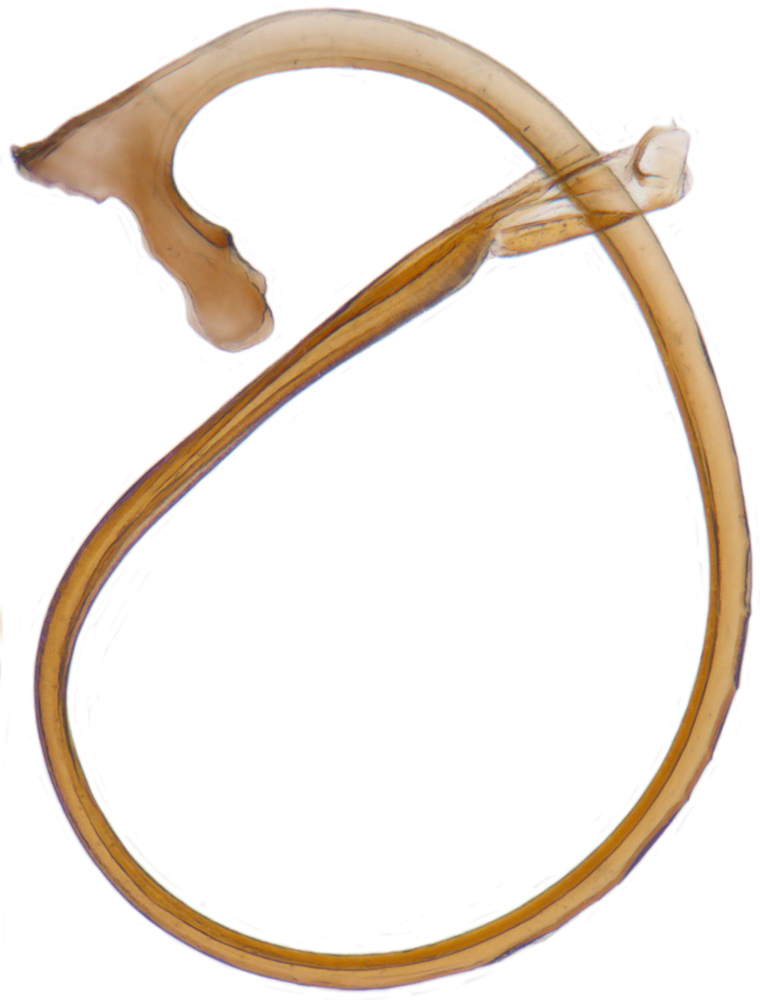
Male genitalia: Penis

**Figure 3a.**
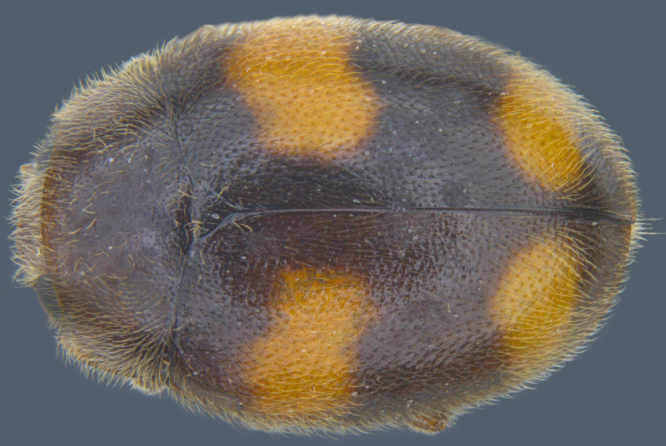
Dorsal view

**Figure 3b.**
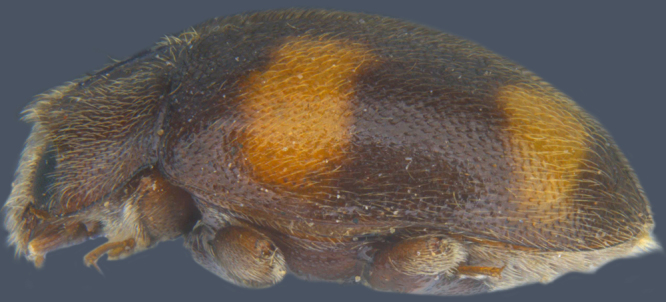
Lateral view

**Figure 4a.**
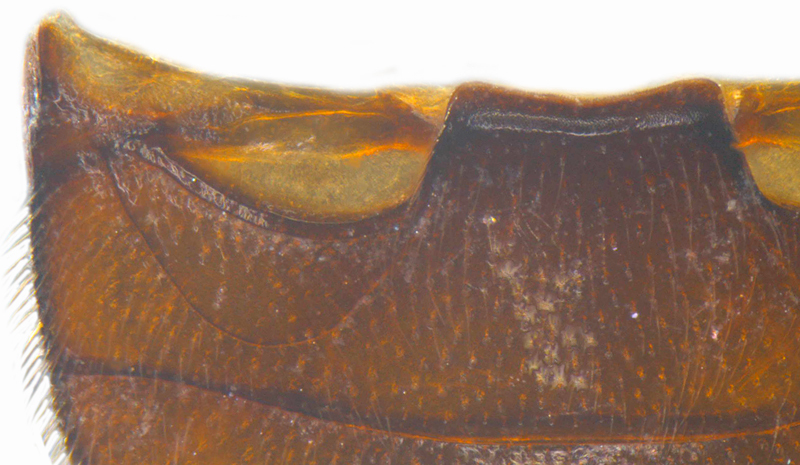
Abdominal postcoxal line

**Figure 4b.**
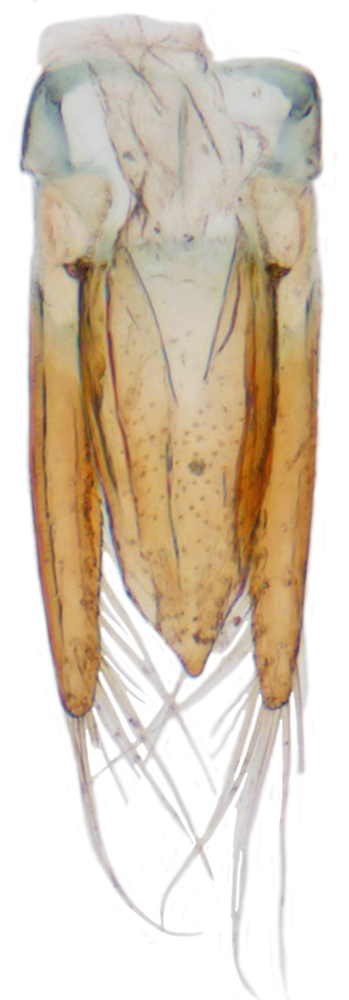
Male genitalia: Tegmen, ventral view

**Figure 4c.**
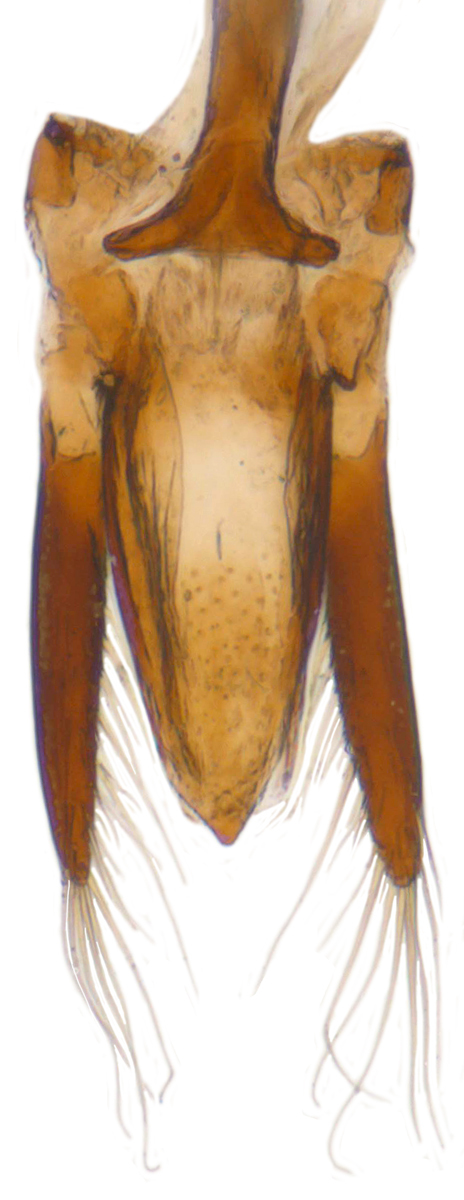
Male genitalia: Tegmen, ventral view

**Figure 4d.**
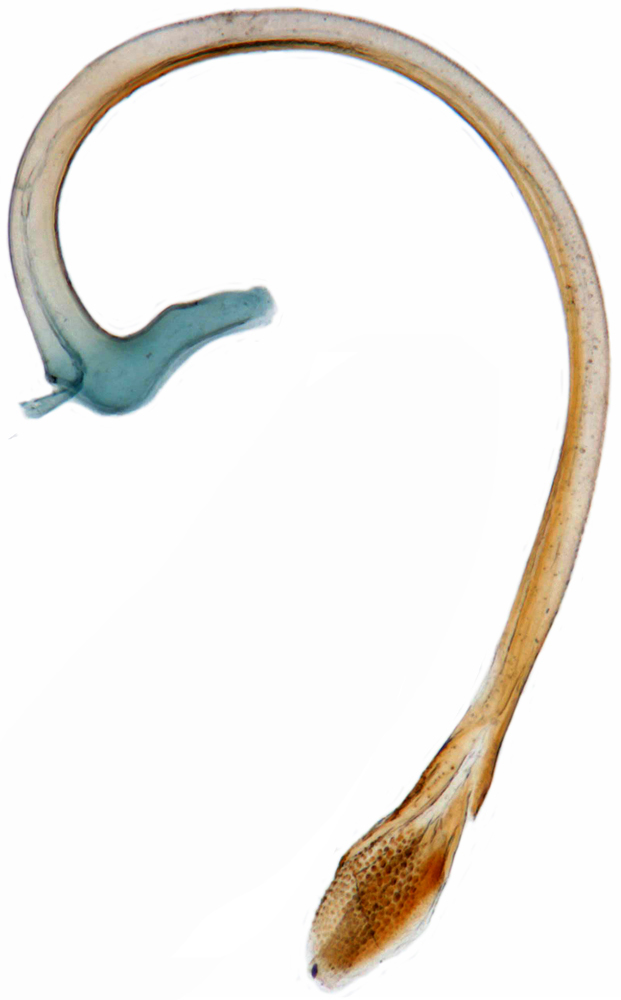
Male genitalia: Penis

**Figure 4e.**
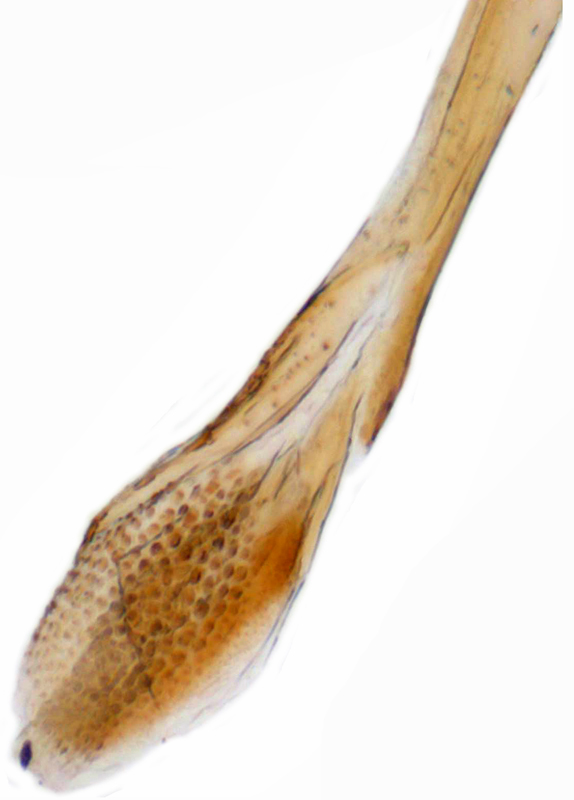
Male genitalia: Penis apex

**Figure 4f.**
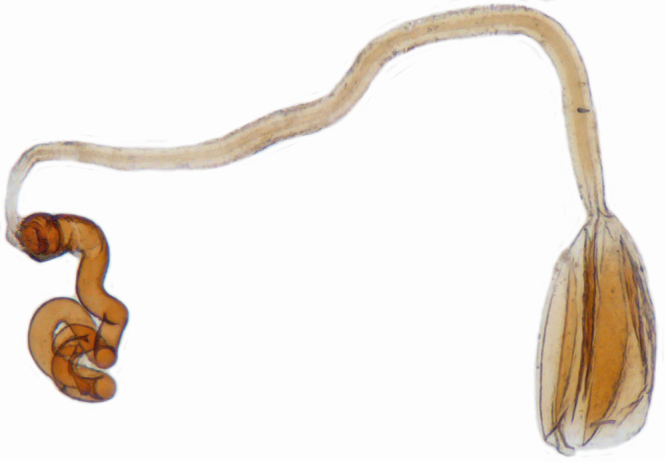
Female genitalia: Spermatheca

**Figure 5a.**
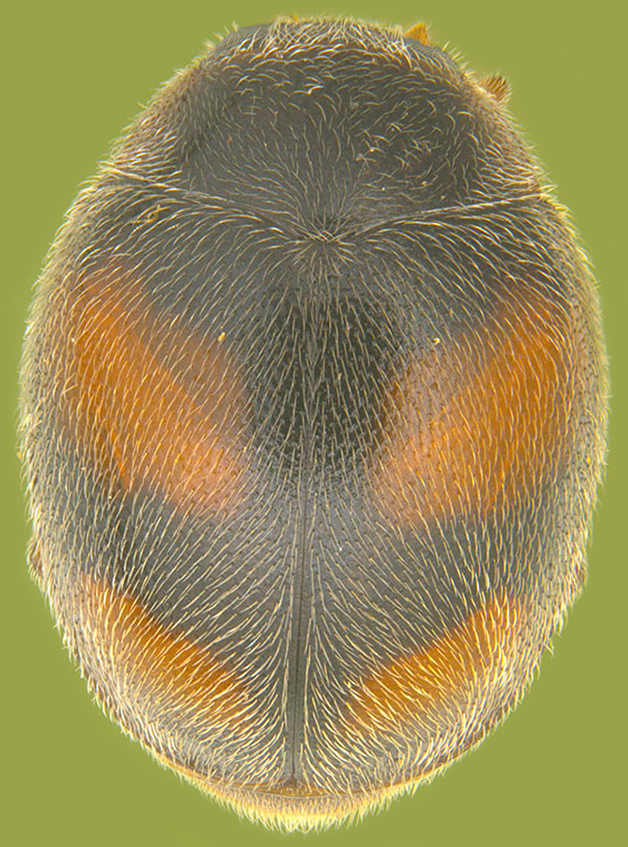
Adult of *S. latemaculatus*, dorsal view

**Figure 5b.**
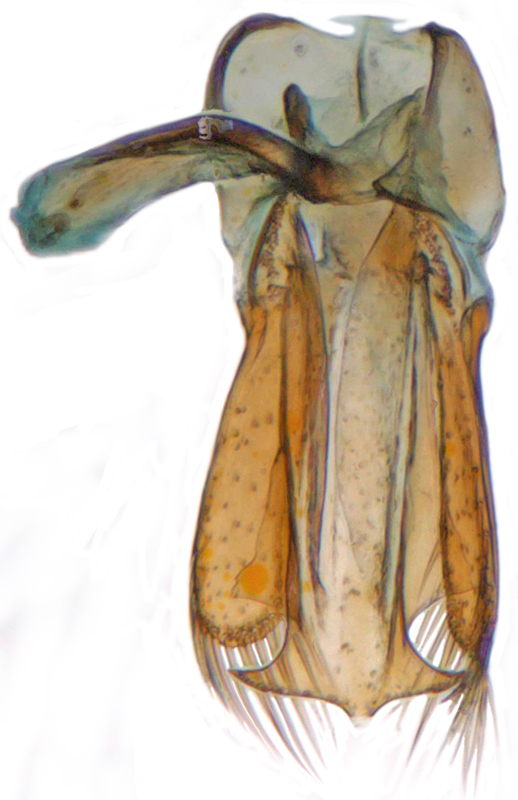
Male genitalia of *S. latemaculatus*: Tegmen, ventral view

**Figure 5c.**
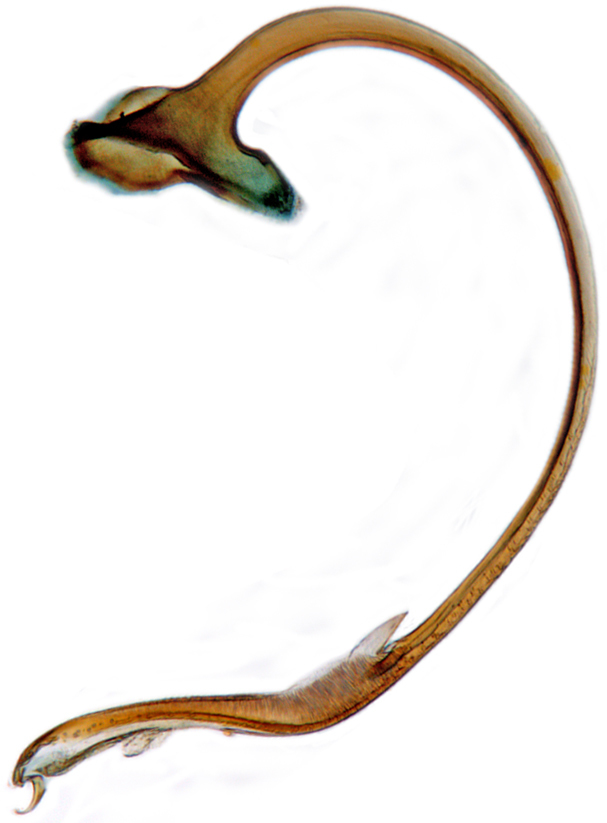
Male genitalia of *S. latemaculatus*: Penis

**Figure 5d.**
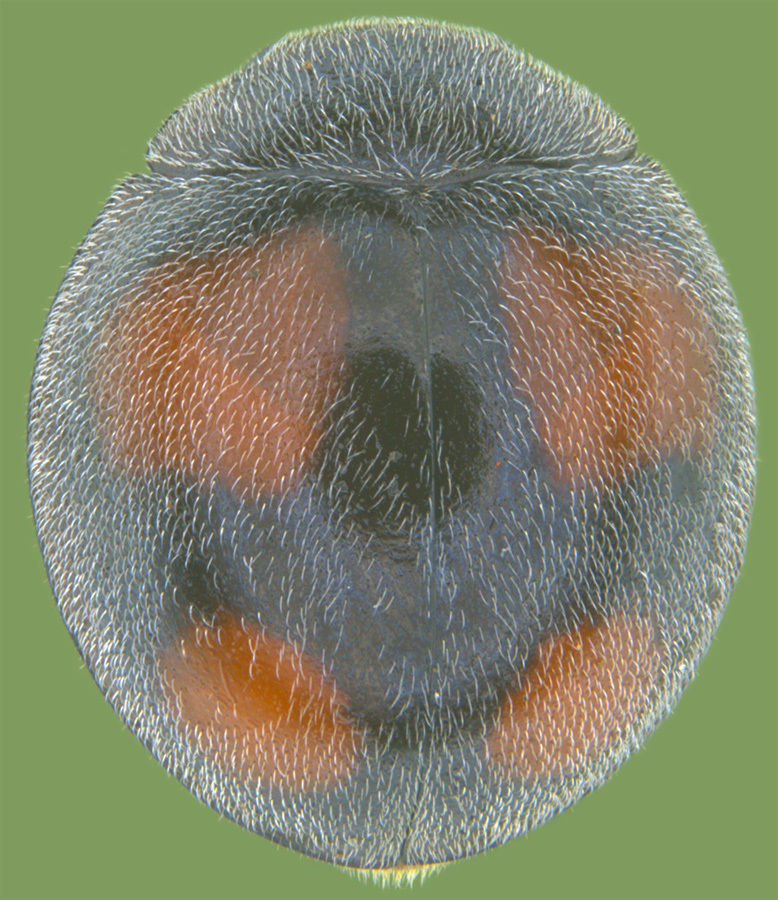
Adult of *P. horni*, dorsal view
